# {(1*R*,3*S*)-2-Benzyl-6,7-dimeth­oxy-1-phenyl-1,2,3,4-tetra­hydro­isoquinolin-3-yl}diphenyl­methanol

**DOI:** 10.1107/S1600536810005295

**Published:** 2010-02-17

**Authors:** Tricia Naicker, Thavendran Govender, Hendrik G. Kruger, Glenn E.M. Maguire

**Affiliations:** a School of Chemistry, University of KwaZulu-Natal, Durban, 4000, South Africa; b School of Pharmacy and Pharmacology, University of KwaZulu-Natal, Durban, 4000, South Africa

## Abstract

In the title compound, C_37_H_35_NO_3_, a precursor to novel chiral catalysts, the N-containing six-membered ring assumes a half-chair conformation. Inter­molecular C—H⋯O hydrogen bonds link the mol­ecules in the crystal structure.

## Related literature

For the synthesis of the title compound, see: Chakka *et al.* (2010 ▶). For related structures, see: Aubry *et al.* (2006 ▶). For a related structure with the same chiral centres and configuration, see: Naicker *et al.* (2009 ▶). For proline diaryl alcohols, see: Diner *et al.* (2008 ▶); Seebach *et al.* (2008 ▶).
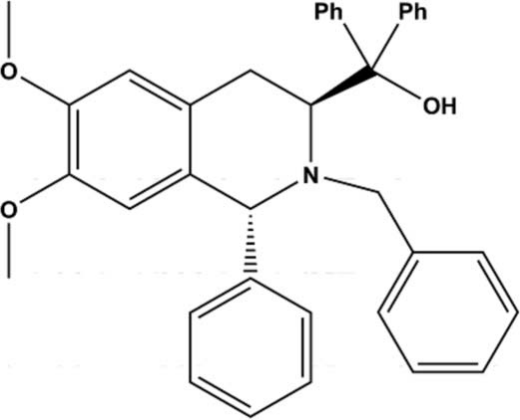


## Experimental

### 

#### Crystal data

C_37_H_35_NO_3_
*M* *_r_* = 541.66
Monoclinic, 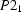

*a* = 11.9706 (5) Å
*b* = 10.1934 (4) Å
*c* = 13.1515 (5) Å
β = 116.546 (2)°
*V* = 1435.58 (10) Å^3^
*Z* = 2
Cu *K*α radiation
μ = 0.62 mm^−1^
*T* = 173 K
0.22 × 0.14 × 0.12 mm

#### Data collection

Bruker Kappa Duo APEXII diffractometer
Absorption correction: multi-scan (*SADABS*; Bruker, 2006 ▶) *T*_min_ = 0.876, *T*_max_ = 0.930
15262 measured reflections
2514 independent reflections
2451 reflections with *I* > 2σ(*I*)
*R*_int_ = 0.025

#### Refinement

*R*[*F* ^2^ > 2σ(*F* ^2^)] = 0.025
*wR*(*F* ^2^) = 0.068
*S* = 1.10
2514 reflections
375 parameters
1 restraint
H atoms treated by a mixture of independent and constrained refinement
Δρ_max_ = 0.14 e Å^−3^
Δρ_min_ = −0.12 e Å^−3^

### 

Data collection: *APEX2* (Bruker, 2006 ▶); cell refinement: *SAINT* (Bruker, 2006 ▶); data reduction: *SAINT*; program(s) used to solve structure: *SHELXS97* (Sheldrick, 2008 ▶); program(s) used to refine structure: *SHELXL97* (Sheldrick, 2008 ▶); molecular graphics: *ORTEP-3* (Farrugia, 1997 ▶) and *DIAMOND* (Brandenburg, 1998 ▶); software used to prepare material for publication: *SHELXL97*.

## Supplementary Material

Crystal structure: contains datablocks I, New_Global_Publ_Block. DOI: 10.1107/S1600536810005295/lx2135sup1.cif

Structure factors: contains datablocks I. DOI: 10.1107/S1600536810005295/lx2135Isup2.hkl

Additional supplementary materials:  crystallographic information; 3D view; checkCIF report

## Figures and Tables

**Table 1:** Hydrogen-bond geometry (Å, °)

*D*—H⋯*A*	*D*—H	H⋯*A*	*D*⋯*A*	*D*—H⋯*A*
C15—H15⋯O2^i^	0.95	2.44	3.385 (2)	171

Symmetry code: (i) 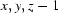.

**Fig. 1.**
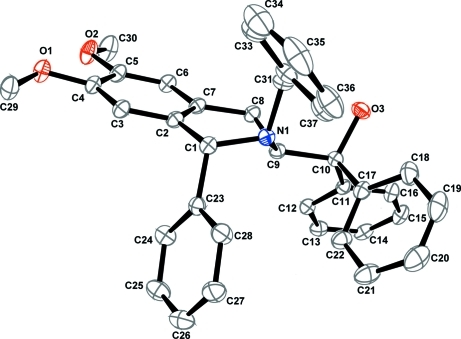
The molecular structure of the title compound with the atom numbering scheme. Displacement ellipsoids are drawn at the 30% probability level. Hydrogen atoms have been omitted for clarity.

**Fig. 2.**
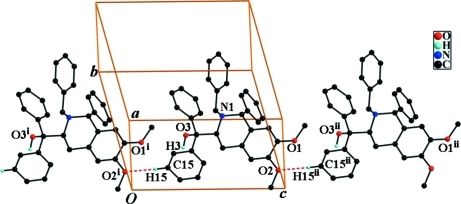
The C—H···O interactions (dotted lines) in the crystal structure of the title compound along the a axis. [Symmetry codes: i) x, y, z -1 ; ii) x, y, z + 1.]

**Fig. 3.**
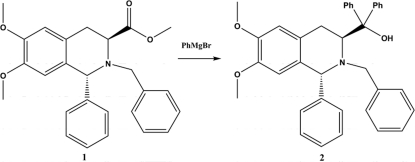
Reaction scheme.

## supplementary crystallographic information

### Comment

The title compound (2, Fig. 3) is a precursor in the synthesis of novel chiral ligands involving a tetrahydroisoquinoline backbone. Recently, we have reported the application of these ligands as useful catalysts for transfer hydrogenation reactions (Chakka *et al.*, 2010).

Compound 2 contains four phenyl rings and the absolute stereochemistry was confirmed to be *R,S* at C1 and C9 positions as shown in Fig. 1, respectively (Aubry *et al.*, 2006). The crystal packing is stabilized by intermolecular C—H···O hydrogen bonds. The H atom of methanol does not form hydrogen bonds (Table 1 & Fig. 2). According to the Cambridge structural data base this is the first tetrahydroisoquinoline derivative with diaryl substitution at the C10 position. The structure displays a *gauche* or sc (synclinal) conformation around the O3—C10—C9—N1 bond with the OH group almost over the piperidine ring with a torsion angle of -77.0 (2)°. Due to the lack of analogous structures this observation was compared to proline diaryl alcohols (Seebach *et al.*, 2008) which display a similar conformation around the exocyclic C9—C10 bond. Given the success of proline diaryl alcohols as a chiral catalyst (Diner *et al.*, 2008) this comparison is particularly useful for catalysts bearing a tetrahydroisoquinoline framework as this feature could have a significant effect on the stereocontrol of the catalyst.

We recently reported a crystal structure of a similar molecule to the title compound (Naicker *et al.*, 2009) which has an ester moiety at the C10 position and the N-containing six membered ring assumes a half boat conformation. The N-containing six membered ring in the title compound exists in a half chair conformation (see Fig. 1). A possible reason for this difference in conformation could be the introduction of large phenyl ring substitiuents at the C10 position. The efficiency of these tetrahydroisoquinoline catalysts is currently being tested in our laboratory.

### Experimental

To a solution of compound 1 (Fig. 3) (500 mg, 1.19 mmol) in THF (10 ml), freshly prepared Grignard reagent of phenyl magnesium bromide (2.17 g, 11.9 mmol) was added under a nitrogen atmosphere at ambient temperature. Completion of the reaction was monitored with TLC by quenching 0.1 ml aliquots of the reaction mixture with saturated ammonium chloride solution at 0 °C using ethyl acetate/hexane as the solvent (40 : 60 R_f_ 0.5). Thereafter the reaction mixture was filtered and the solvent was evaporated under reduced pressure to afford the crude product. This was purified by column chromatography using ethyl acetate/hexane (40:60) as the eluent to yield 80 % (0.52 g) pure tetrahydroisoquinoline diphenyl alcohol 2 as a white solid. ^1^H NMR (600 MHz,CDCl_3_,δ, p.p.m): 7.38 (d, *J *= 7.26 Hz, 2H), 7.32–7.16 (m, 9H), 6.54 (s, 1H), 6.26 (s, 1H), 5.19 (s, 1H), 3.85–3.72 (m, 6H), 3.61 (s,6*H*), 3.23 (dd, *J* = 5.10, 15.66 Hz, 1H), 2.98 (dd, *J* = 3.00, 15.72, Hz, 1H). Light yellow crystals suitable for X-ray diffraction were obtained by slow evaporation of 2 in dichloromethane at room temperature.

### Refinement

The H atom of O3 was located in difference Fourier map and freely refined. All H atoms and C atoms were positioned geometrically and refined using a riding model, with C—H = 1.00 (CH), 0.99 (CH_2_), 0.98 (CH_3_) and 0.93 (aromatic CH) Å. *U*_iso_(H) = 1.5*U*_eq_ (C) for methyl H atoms and 1.2*U*_eq_(C) for other all H atoms. In the absence of significant anomalous scattering effects, Friedel pairs were merged.

### Crystal data

**Table:**

C_37_H_35_NO_3_	*F*(000) = 576
*M**_r_* = 541.66	*D*_x_ = 1.253 Mg m^−^^3^
Monoclinic, *P*2_1_	Melting point: 478 K
Hall symbol: P 2yb	Cu *K*α radiation, λ = 1.54184 Å
*a* = 11.9706 (5) Å	Cell parameters from 15260 reflections
*b* = 10.1934 (4) Å	θ = 4.1–64.1°
*c* = 13.1515 (5) Å	µ = 0.62 mm^−^^1^
β = 116.546 (2)°	*T* = 173 K
*V* = 1435.58 (10) Å^3^	Needle, light-yellow
*Z* = 2	0.22 × 0.14 × 0.12 mm

### Data collection

**Table:**

Bruker Kappa Duo APEXII diffractometer	2514 independent reflections
Radiation source: fine-focus sealed tube	2451 reflections with *I* > 2σ(*I*)
graphite	*R*_int_ = 0.025
0.5° φ scans and ω scans	θ_max_ = 64.1°, θ_min_ = 4.1°
Absorption correction: multi-scan (*SADABS*; Bruker, 2006)	*h* = −13→13
*T*_min_ = 0.876, *T*_max_ = 0.930	*k* = −11→11
15262 measured reflections	*l* = −15→15

### Refinement

**Table:**

Refinement on *F*^2^	Secondary atom site location: difference Fourier map
Least-squares matrix: full	Hydrogen site location: difference Fourier map
*R*[*F*^2^ > 2σ(*F*^2^)] = 0.025	H atoms treated by a mixture of independent and constrained refinement
*wR*(*F*^2^) = 0.068	*w* = 1/[σ^2^(*F*_o_^2^) + (0.0394*P*)^2^ + 0.1556*P*] where *P* = (*F*_o_^2^ + 2*F*_c_^2^)/3
*S* = 1.10	(Δ/σ)_max_ < 0.001
2514 reflections	Δρ_max_ = 0.14 e Å^−^^3^
375 parameters	Δρ_min_ = −0.12 e Å^−^^3^
1 restraint	Extinction correction: *SHELXL*, Fc^*^=kFc[1+0.001xFc^2^λ^3^/sin(2θ)]^-1/4^
Primary atom site location: structure-invariant direct methods	Extinction coefficient: 0.0019 (4)

### Special details

**Table:**

Experimental. Half sphere of data collected using SAINT strategy (Bruker, 2006). Crystal to detector distance = 50 mm; combination of φ and ω scans of 0.5°, 70 s per °, 2 iterations.
Geometry. All esds (except the esd in the dihedral angle between two l.s. planes) are estimated using the full covariance matrix. The cell esds are taken into account individually in the estimation of esds in distances, angles and torsion angles; correlations between esds in cell parameters are only used when they are defined by crystal symmetry. An approximate (isotropic) treatment of cell esds is used for estimating esds involving l.s. planes.
Refinement. Refinement of *F*^2^ against ALL reflections. The weighted *R*-factor wR and goodness of fit *S* are based on *F*^2^, conventional *R*-factors *R* are based on *F*, with *F* set to zero for negative *F*^2^. The threshold expression of *F*^2^ > σ(*F*^2^) is used only for calculating *R*-factors(gt) etc. and is not relevant to the choice of reflections for refinement. *R*-factors based on *F*^2^ are statistically about twice as large as those based on *F*, and *R*- factors based on ALL data will be even larger.

### Fractional atomic coordinates and isotropic or equivalent isotropic displacement parameters (Å^2^)

**Table:**

	*x*	*y*	*z*	*U*_iso_*/*U*_eq_	
O1	0.22854 (13)	0.26734 (16)	1.12303 (10)	0.0431 (3)	
O2	0.04675 (14)	0.12697 (17)	0.98256 (12)	0.0500 (4)	
O3	0.05661 (12)	0.42417 (16)	0.44410 (11)	0.0404 (3)	
H3O	−0.004 (3)	0.362 (3)	0.413 (2)	0.075 (9)*	
N1	0.25806 (13)	0.46610 (15)	0.69414 (12)	0.0293 (3)	
C1	0.32465 (16)	0.43732 (19)	0.81717 (14)	0.0301 (4)	
H1	0.3345	0.5232	0.8571	0.036*	
C2	0.24903 (16)	0.34980 (18)	0.85715 (15)	0.0308 (4)	
C3	0.27894 (17)	0.34611 (19)	0.97314 (15)	0.0329 (4)	
H3	0.3486	0.3948	1.0255	0.040*	
C4	0.20987 (17)	0.2737 (2)	1.01278 (15)	0.0346 (4)	
C5	0.10937 (17)	0.1980 (2)	0.93545 (16)	0.0366 (4)	
C6	0.08020 (17)	0.2006 (2)	0.82194 (15)	0.0348 (4)	
H6	0.0123	0.1494	0.7702	0.042*	
C7	0.14841 (16)	0.27720 (19)	0.78076 (15)	0.0315 (4)	
C8	0.10892 (16)	0.2823 (2)	0.65460 (15)	0.0327 (4)	
H8A	0.0316	0.3350	0.6169	0.039*	
H8B	0.0906	0.1924	0.6229	0.039*	
C9	0.21113 (16)	0.34278 (18)	0.63028 (14)	0.0294 (4)	
H9	0.2827	0.2798	0.6612	0.035*	
C10	0.17522 (16)	0.35837 (19)	0.50136 (15)	0.0310 (4)	
C11	0.16612 (15)	0.22675 (19)	0.44078 (15)	0.0307 (4)	
C12	0.19679 (17)	0.1051 (2)	0.49339 (15)	0.0339 (4)	
H12	0.2266	0.0994	0.5734	0.041*	
C13	0.18466 (18)	−0.0085 (2)	0.43084 (18)	0.0384 (4)	
H13	0.2054	−0.0910	0.4683	0.046*	
C14	0.14278 (19)	−0.0023 (2)	0.31498 (18)	0.0409 (5)	
H14	0.1338	−0.0802	0.2724	0.049*	
C15	0.11407 (19)	0.1175 (2)	0.26129 (16)	0.0434 (5)	
H15	0.0862	0.1227	0.1815	0.052*	
C16	0.12580 (18)	0.2302 (2)	0.32337 (16)	0.0394 (5)	
H16	0.1059	0.3123	0.2853	0.047*	
C17	0.27291 (17)	0.44212 (19)	0.48694 (14)	0.0334 (4)	
C18	0.2411 (2)	0.5509 (2)	0.41693 (18)	0.0489 (5)	
H18	0.1563	0.5780	0.3789	0.059*	
C19	0.3321 (3)	0.6211 (3)	0.4016 (2)	0.0626 (7)	
H19	0.3091	0.6952	0.3527	0.075*	
C20	0.4552 (3)	0.5837 (3)	0.4569 (2)	0.0636 (7)	
H20	0.5172	0.6317	0.4463	0.076*	
C21	0.4880 (2)	0.4767 (3)	0.5273 (2)	0.0553 (6)	
H21	0.5729	0.4504	0.5656	0.066*	
C22	0.39822 (18)	0.4074 (2)	0.54256 (18)	0.0409 (5)	
H22	0.4224	0.3340	0.5923	0.049*	
C23	0.45677 (16)	0.38930 (19)	0.84700 (14)	0.0314 (4)	
C24	0.50143 (18)	0.2662 (2)	0.89154 (16)	0.0392 (4)	
H24	0.4496	0.2076	0.9075	0.047*	
C25	0.6210 (2)	0.2279 (2)	0.9130 (2)	0.0513 (5)	
H25	0.6500	0.1428	0.9424	0.062*	
C26	0.6982 (2)	0.3123 (3)	0.8920 (2)	0.0532 (6)	
H26	0.7797	0.2852	0.9058	0.064*	
C27	0.65631 (19)	0.4367 (3)	0.85060 (19)	0.0526 (6)	
H27	0.7097	0.4966	0.8381	0.063*	
C28	0.53601 (18)	0.4734 (2)	0.82747 (18)	0.0447 (5)	
H28	0.5071	0.5583	0.7975	0.054*	
C29	0.3369 (2)	0.3293 (3)	1.20594 (16)	0.0490 (5)	
H29A	0.3399	0.3181	1.2811	0.074*	
H29B	0.3344	0.4230	1.1885	0.074*	
H29C	0.4112	0.2896	1.2055	0.074*	
C30	−0.0409 (2)	0.0329 (3)	0.9135 (2)	0.0639 (7)	
H30A	−0.0784	−0.0106	0.9573	0.096*	
H30B	0.0012	−0.0324	0.8880	0.096*	
H30C	−0.1064	0.0765	0.8473	0.096*	
C31	0.16184 (17)	0.5677 (2)	0.67155 (17)	0.0359 (4)	
H31A	0.1115	0.5449	0.7117	0.043*	
H31B	0.1052	0.5709	0.5891	0.043*	
C32	0.22097 (17)	0.7003 (2)	0.71081 (16)	0.0391 (5)	
C33	0.1997 (2)	0.7730 (3)	0.7892 (2)	0.0552 (6)	
H33	0.1479	0.7391	0.8206	0.066*	
C34	0.2552 (3)	0.8977 (3)	0.8222 (2)	0.0750 (9)	
H34	0.2416	0.9479	0.8765	0.090*	
C35	0.3287 (3)	0.9462 (3)	0.7760 (2)	0.0765 (9)	
H35	0.3647	1.0310	0.7973	0.092*	
C36	0.3507 (2)	0.8743 (3)	0.6998 (2)	0.0637 (7)	
H36	0.4025	0.9088	0.6686	0.076*	
C37	0.2985 (2)	0.7520 (2)	0.66755 (19)	0.0495 (5)	
H37	0.3156	0.7021	0.6151	0.059*	

### Atomic displacement parameters (Å^2^)

**Table:**

	*U*^11^	*U*^22^	*U*^33^	*U*^12^	*U*^13^	*U*^23^
O1	0.0513 (8)	0.0493 (8)	0.0319 (6)	−0.0119 (7)	0.0214 (6)	−0.0048 (6)
O2	0.0593 (9)	0.0570 (10)	0.0414 (7)	−0.0261 (8)	0.0294 (7)	−0.0056 (7)
O3	0.0328 (7)	0.0446 (8)	0.0367 (7)	0.0091 (7)	0.0094 (5)	0.0015 (7)
N1	0.0304 (7)	0.0272 (8)	0.0309 (7)	−0.0005 (6)	0.0143 (6)	0.0002 (6)
C1	0.0332 (9)	0.0264 (9)	0.0315 (8)	−0.0034 (8)	0.0154 (7)	−0.0028 (8)
C2	0.0342 (9)	0.0278 (9)	0.0335 (9)	−0.0016 (8)	0.0179 (7)	−0.0032 (8)
C3	0.0372 (9)	0.0308 (10)	0.0325 (8)	−0.0048 (8)	0.0171 (7)	−0.0061 (8)
C4	0.0431 (10)	0.0336 (10)	0.0317 (8)	−0.0012 (9)	0.0209 (8)	−0.0022 (8)
C5	0.0419 (10)	0.0360 (11)	0.0384 (9)	−0.0066 (9)	0.0239 (8)	−0.0022 (9)
C6	0.0363 (9)	0.0347 (10)	0.0359 (9)	−0.0082 (8)	0.0185 (8)	−0.0058 (8)
C7	0.0341 (9)	0.0292 (9)	0.0348 (9)	−0.0022 (8)	0.0184 (7)	−0.0037 (8)
C8	0.0340 (9)	0.0334 (10)	0.0324 (9)	−0.0038 (8)	0.0164 (7)	−0.0041 (8)
C9	0.0306 (8)	0.0272 (9)	0.0320 (9)	0.0002 (7)	0.0153 (7)	−0.0010 (7)
C10	0.0299 (9)	0.0320 (10)	0.0321 (9)	0.0035 (8)	0.0146 (7)	0.0032 (8)
C11	0.0274 (8)	0.0360 (10)	0.0326 (9)	−0.0043 (8)	0.0169 (7)	−0.0012 (8)
C12	0.0354 (9)	0.0362 (10)	0.0313 (9)	0.0015 (8)	0.0160 (7)	−0.0006 (8)
C13	0.0367 (10)	0.0337 (11)	0.0480 (11)	0.0005 (8)	0.0218 (9)	−0.0014 (9)
C14	0.0415 (10)	0.0444 (12)	0.0462 (11)	−0.0106 (9)	0.0281 (9)	−0.0145 (9)
C15	0.0504 (11)	0.0527 (13)	0.0334 (9)	−0.0138 (11)	0.0245 (9)	−0.0082 (10)
C16	0.0465 (10)	0.0420 (12)	0.0331 (9)	−0.0069 (9)	0.0208 (8)	0.0017 (9)
C17	0.0421 (10)	0.0290 (10)	0.0332 (8)	−0.0022 (8)	0.0206 (8)	−0.0022 (8)
C18	0.0616 (13)	0.0418 (12)	0.0446 (11)	0.0023 (11)	0.0248 (10)	0.0105 (10)
C19	0.100 (2)	0.0388 (13)	0.0616 (14)	−0.0096 (14)	0.0470 (14)	0.0111 (12)
C20	0.0764 (18)	0.0510 (16)	0.0845 (18)	−0.0174 (13)	0.0548 (15)	0.0012 (14)
C21	0.0490 (12)	0.0551 (15)	0.0755 (16)	−0.0087 (12)	0.0401 (12)	−0.0028 (13)
C22	0.0422 (10)	0.0355 (11)	0.0525 (11)	0.0002 (9)	0.0278 (9)	0.0036 (9)
C23	0.0319 (9)	0.0327 (10)	0.0274 (8)	−0.0035 (8)	0.0113 (7)	−0.0033 (7)
C24	0.0394 (10)	0.0300 (10)	0.0444 (10)	−0.0043 (9)	0.0154 (8)	−0.0038 (9)
C25	0.0447 (11)	0.0395 (12)	0.0604 (13)	0.0066 (10)	0.0151 (10)	−0.0008 (10)
C26	0.0353 (10)	0.0587 (16)	0.0612 (14)	0.0057 (11)	0.0174 (10)	−0.0032 (12)
C27	0.0374 (11)	0.0624 (16)	0.0598 (13)	−0.0038 (11)	0.0233 (10)	0.0089 (13)
C28	0.0377 (10)	0.0435 (12)	0.0524 (11)	−0.0004 (9)	0.0197 (9)	0.0118 (10)
C29	0.0525 (12)	0.0600 (14)	0.0337 (10)	−0.0104 (11)	0.0185 (9)	−0.0055 (10)
C30	0.0733 (16)	0.0710 (18)	0.0550 (13)	−0.0382 (15)	0.0354 (12)	−0.0082 (13)
C31	0.0318 (9)	0.0307 (10)	0.0434 (10)	0.0024 (8)	0.0152 (8)	−0.0016 (8)
C32	0.0346 (9)	0.0282 (10)	0.0416 (10)	0.0059 (8)	0.0057 (8)	0.0012 (9)
C33	0.0553 (13)	0.0428 (13)	0.0526 (12)	0.0115 (11)	0.0108 (10)	−0.0074 (11)
C34	0.093 (2)	0.0450 (16)	0.0549 (14)	0.0174 (15)	0.0041 (14)	−0.0155 (12)
C35	0.0830 (19)	0.0335 (14)	0.0658 (16)	−0.0077 (13)	−0.0091 (14)	0.0039 (13)
C36	0.0584 (14)	0.0397 (13)	0.0640 (15)	−0.0079 (11)	0.0014 (11)	0.0134 (12)
C37	0.0462 (11)	0.0364 (12)	0.0537 (12)	0.0007 (10)	0.0113 (9)	0.0092 (10)

### Geometric parameters (Å, °)

**Table:**

O1—C4	1.366 (2)	C18—C19	1.392 (3)
O1—C29	1.416 (3)	C18—H18	0.9500
O2—C5	1.373 (2)	C19—C20	1.374 (4)
O2—C30	1.412 (3)	C19—H19	0.9500
O3—C10	1.441 (2)	C20—C21	1.370 (4)
O3—H3O	0.91 (3)	C20—H20	0.9500
N1—C9	1.476 (2)	C21—C22	1.373 (3)
N1—C31	1.477 (2)	C21—H21	0.9500
N1—C1	1.479 (2)	C22—H22	0.9500
C1—C2	1.522 (2)	C23—C28	1.385 (3)
C1—C23	1.530 (2)	C23—C24	1.387 (3)
C1—H1	1.0000	C24—C25	1.386 (3)
C2—C7	1.388 (3)	C24—H24	0.9500
C2—C3	1.403 (2)	C25—C26	1.378 (3)
C3—C4	1.373 (3)	C25—H25	0.9500
C3—H3	0.9500	C26—C27	1.382 (4)
C4—C5	1.408 (3)	C26—H26	0.9500
C5—C6	1.373 (3)	C27—C28	1.384 (3)
C6—C7	1.402 (3)	C27—H27	0.9500
C6—H6	0.9500	C28—H28	0.9500
C7—C8	1.509 (2)	C29—H29A	0.9800
C8—C9	1.526 (2)	C29—H29B	0.9800
C8—H8A	0.9900	C29—H29C	0.9800
C8—H8B	0.9900	C30—H30A	0.9800
C9—C10	1.560 (2)	C30—H30B	0.9800
C9—H9	1.0000	C30—H30C	0.9800
C10—C17	1.527 (3)	C31—C32	1.506 (3)
C10—C11	1.539 (3)	C31—H31A	0.9900
C11—C12	1.387 (3)	C31—H31B	0.9900
C11—C16	1.397 (3)	C32—C33	1.382 (3)
C12—C13	1.390 (3)	C32—C37	1.391 (3)
C12—H12	0.9500	C33—C34	1.410 (4)
C13—C14	1.376 (3)	C33—H33	0.9500
C13—H13	0.9500	C34—C35	1.365 (5)
C14—C15	1.375 (3)	C34—H34	0.9500
C14—H14	0.9500	C35—C36	1.360 (4)
C15—C16	1.379 (3)	C35—H35	0.9500
C15—H15	0.9500	C36—C37	1.373 (3)
C16—H16	0.9500	C36—H36	0.9500
C17—C18	1.382 (3)	C37—H37	0.9500
C17—C22	1.389 (3)		
			
C4—O1—C29	117.08 (15)	C17—C18—C19	120.6 (2)
C5—O2—C30	117.80 (15)	C17—C18—H18	119.7
C10—O3—H3O	108 (2)	C19—C18—H18	119.7
C9—N1—C31	114.74 (13)	C20—C19—C18	120.3 (2)
C9—N1—C1	109.60 (14)	C20—C19—H19	119.8
C31—N1—C1	110.47 (14)	C18—C19—H19	119.8
N1—C1—C2	112.41 (14)	C21—C20—C19	119.6 (2)
N1—C1—C23	109.51 (13)	C21—C20—H20	120.2
C2—C1—C23	115.48 (15)	C19—C20—H20	120.2
N1—C1—H1	106.3	C20—C21—C22	120.2 (2)
C2—C1—H1	106.3	C20—C21—H21	119.9
C23—C1—H1	106.3	C22—C21—H21	119.9
C7—C2—C3	119.34 (16)	C21—C22—C17	121.5 (2)
C7—C2—C1	121.35 (15)	C21—C22—H22	119.2
C3—C2—C1	119.28 (16)	C17—C22—H22	119.2
C4—C3—C2	121.52 (17)	C28—C23—C24	118.13 (18)
C4—C3—H3	119.2	C28—C23—C1	118.02 (17)
C2—C3—H3	119.2	C24—C23—C1	123.85 (17)
O1—C4—C3	125.99 (17)	C25—C24—C23	120.6 (2)
O1—C4—C5	114.97 (16)	C25—C24—H24	119.7
C3—C4—C5	119.04 (16)	C23—C24—H24	119.7
C6—C5—O2	125.18 (17)	C26—C25—C24	120.5 (2)
C6—C5—C4	119.63 (16)	C26—C25—H25	119.7
O2—C5—C4	115.18 (16)	C24—C25—H25	119.7
C5—C6—C7	121.47 (17)	C25—C26—C27	119.6 (2)
C5—C6—H6	119.3	C25—C26—H26	120.2
C7—C6—H6	119.3	C27—C26—H26	120.2
C2—C7—C6	118.96 (16)	C26—C27—C28	119.6 (2)
C2—C7—C8	121.43 (16)	C26—C27—H27	120.2
C6—C7—C8	119.58 (15)	C28—C27—H27	120.2
C7—C8—C9	110.96 (14)	C27—C28—C23	121.6 (2)
C7—C8—H8A	109.4	C27—C28—H28	119.2
C9—C8—H8A	109.4	C23—C28—H28	119.2
C7—C8—H8B	109.4	O1—C29—H29A	109.5
C9—C8—H8B	109.4	O1—C29—H29B	109.5
H8A—C8—H8B	108.0	H29A—C29—H29B	109.5
N1—C9—C8	111.20 (14)	O1—C29—H29C	109.5
N1—C9—C10	112.43 (15)	H29A—C29—H29C	109.5
C8—C9—C10	114.31 (14)	H29B—C29—H29C	109.5
N1—C9—H9	106.1	O2—C30—H30A	109.5
C8—C9—H9	106.1	O2—C30—H30B	109.5
C10—C9—H9	106.1	H30A—C30—H30B	109.5
O3—C10—C17	107.77 (15)	O2—C30—H30C	109.5
O3—C10—C11	108.05 (14)	H30A—C30—H30C	109.5
C17—C10—C11	107.71 (14)	H30B—C30—H30C	109.5
O3—C10—C9	110.11 (14)	N1—C31—C32	110.84 (14)
C17—C10—C9	109.74 (14)	N1—C31—H31A	109.5
C11—C10—C9	113.28 (15)	C32—C31—H31A	109.5
C12—C11—C16	117.22 (18)	N1—C31—H31B	109.5
C12—C11—C10	125.39 (15)	C32—C31—H31B	109.5
C16—C11—C10	117.37 (17)	H31A—C31—H31B	108.1
C11—C12—C13	121.01 (16)	C33—C32—C37	118.8 (2)
C11—C12—H12	119.5	C33—C32—C31	121.4 (2)
C13—C12—H12	119.5	C37—C32—C31	119.80 (19)
C14—C13—C12	120.46 (19)	C32—C33—C34	119.6 (3)
C14—C13—H13	119.8	C32—C33—H33	120.2
C12—C13—H13	119.8	C34—C33—H33	120.2
C13—C14—C15	119.53 (19)	C35—C34—C33	119.9 (3)
C13—C14—H14	120.2	C35—C34—H34	120.1
C15—C14—H14	120.2	C33—C34—H34	120.1
C14—C15—C16	119.97 (17)	C36—C35—C34	120.6 (3)
C14—C15—H15	120.0	C36—C35—H35	119.7
C16—C15—H15	120.0	C34—C35—H35	119.7
C15—C16—C11	121.8 (2)	C35—C36—C37	120.4 (3)
C15—C16—H16	119.1	C35—C36—H36	119.8
C11—C16—H16	119.1	C37—C36—H36	119.8
C18—C17—C22	117.82 (18)	C36—C37—C32	120.8 (3)
C18—C17—C10	122.22 (17)	C36—C37—H37	119.6
C22—C17—C10	119.94 (17)	C32—C37—H37	119.6
			
C9—N1—C1—C2	−49.61 (18)	C16—C11—C12—C13	−1.6 (3)
C31—N1—C1—C2	77.77 (18)	C10—C11—C12—C13	179.67 (18)
C9—N1—C1—C23	80.17 (17)	C11—C12—C13—C14	0.6 (3)
C31—N1—C1—C23	−152.45 (15)	C12—C13—C14—C15	0.7 (3)
N1—C1—C2—C7	17.4 (2)	C13—C14—C15—C16	−0.8 (3)
C23—C1—C2—C7	−109.28 (19)	C14—C15—C16—C11	−0.2 (3)
N1—C1—C2—C3	−160.34 (16)	C12—C11—C16—C15	1.4 (3)
C23—C1—C2—C3	73.0 (2)	C10—C11—C16—C15	−179.74 (17)
C7—C2—C3—C4	−0.9 (3)	O3—C10—C17—C18	−7.7 (2)
C1—C2—C3—C4	176.84 (17)	C11—C10—C17—C18	108.7 (2)
C29—O1—C4—C3	−7.4 (3)	C9—C10—C17—C18	−127.62 (19)
C29—O1—C4—C5	172.86 (19)	O3—C10—C17—C22	174.30 (16)
C2—C3—C4—O1	−177.54 (18)	C11—C10—C17—C22	−69.3 (2)
C2—C3—C4—C5	2.2 (3)	C9—C10—C17—C22	54.4 (2)
C30—O2—C5—C6	11.0 (3)	C22—C17—C18—C19	1.2 (3)
C30—O2—C5—C4	−169.7 (2)	C10—C17—C18—C19	−176.8 (2)
O1—C4—C5—C6	178.01 (18)	C17—C18—C19—C20	−0.6 (4)
C3—C4—C5—C6	−1.8 (3)	C18—C19—C20—C21	0.0 (4)
O1—C4—C5—O2	−1.3 (3)	C19—C20—C21—C22	−0.1 (4)
C3—C4—C5—O2	178.95 (18)	C20—C21—C22—C17	0.7 (3)
O2—C5—C6—C7	179.27 (19)	C18—C17—C22—C21	−1.3 (3)
C4—C5—C6—C7	0.0 (3)	C10—C17—C22—C21	176.81 (19)
C3—C2—C7—C6	−0.8 (3)	N1—C1—C23—C28	61.0 (2)
C1—C2—C7—C6	−178.51 (17)	C2—C1—C23—C28	−170.93 (16)
C3—C2—C7—C8	177.34 (17)	N1—C1—C23—C24	−118.47 (18)
C1—C2—C7—C8	−0.4 (3)	C2—C1—C23—C24	9.6 (2)
C5—C6—C7—C2	1.2 (3)	C28—C23—C24—C25	−1.6 (3)
C5—C6—C7—C8	−176.95 (18)	C1—C23—C24—C25	177.84 (18)
C2—C7—C8—C9	15.5 (2)	C23—C24—C25—C26	1.0 (3)
C6—C7—C8—C9	−166.40 (17)	C24—C25—C26—C27	0.9 (4)
C31—N1—C9—C8	−57.74 (19)	C25—C26—C27—C28	−2.0 (4)
C1—N1—C9—C8	67.21 (17)	C26—C27—C28—C23	1.4 (4)
C31—N1—C9—C10	71.89 (18)	C24—C23—C28—C27	0.4 (3)
C1—N1—C9—C10	−163.16 (14)	C1—C23—C28—C27	−179.1 (2)
C7—C8—C9—N1	−48.4 (2)	C9—N1—C31—C32	−162.95 (15)
C7—C8—C9—C10	−177.03 (16)	C1—N1—C31—C32	72.56 (19)
N1—C9—C10—O3	−77.04 (18)	N1—C31—C32—C33	−122.4 (2)
C8—C9—C10—O3	51.0 (2)	N1—C31—C32—C37	57.8 (2)
N1—C9—C10—C17	41.44 (19)	C37—C32—C33—C34	0.8 (3)
C8—C9—C10—C17	169.44 (15)	C31—C32—C33—C34	−178.93 (19)
N1—C9—C10—C11	161.84 (14)	C32—C33—C34—C35	0.5 (4)
C8—C9—C10—C11	−70.15 (19)	C33—C34—C35—C36	−1.2 (4)
O3—C10—C11—C12	−127.94 (18)	C34—C35—C36—C37	0.5 (4)
C17—C10—C11—C12	115.89 (18)	C35—C36—C37—C32	0.9 (3)
C9—C10—C11—C12	−5.7 (2)	C33—C32—C37—C36	−1.6 (3)
O3—C10—C11—C16	53.3 (2)	C31—C32—C37—C36	178.20 (18)
C17—C10—C11—C16	−62.88 (19)	O3—C10—C9—N1	−77.04 (18)
C9—C10—C11—C16	175.57 (15)		

### Hydrogen-bond geometry (Å, °)

**Table:**

*D*—H···*A*	*D*—H	H···*A*	*D*···*A*	*D*—H···*A*
C15—H15···O2^i^	0.95	2.44	3.385 (2)	171

Symmetry codes: (i) *x*, *y*, *z*−1.
